# Supplementary material for: MgAl-Layered-Double-Hydroxide/Sepiolite Composite Membrane for High-Performance Water Treatment Based on Layer-by-Layer Hierarchical Architectures
Source: Polymers (Basel). 2019 Mar 20;11(3):525. doi: 10.3390/polym11030525 (PMC6473615; doi:10.3390/polym11030525)
Supplement: Supplementary file 1 [file polymers-11-00525-s001.pdf]

# Supporting information

MgAl-Layered-Double-Hydroxide/sepiolite composite membrane for high-performance water treatment based on layer-by-layer hierarchical architectures

Zongxue Yu [a,b,c*] Xiuhui Li [a,b] Yixin Peng [a,b] Xia Min [a,b] Di Yin [a,b] Yanliang Shao [a,b]

*(a. College of Chemistry and Chemical Engineering, Southwest Petroleum University, Chengdu, Sichuan 610500, PR of China; b. Oil & Gas Field Applied Chemistry Key Laboratory of Sichuan Province, Southwest Petroleum University. Chengdu, Sichuan 610500, P R of China; c. State Key Laboratory of Oil & Gas Reservoir Geology and Exploitation, Southwest Petroleum University, Chengdu, Sichuan 610500, P R of China)*Address correspondence to this author. Zongxue Yu: Email: haiqingy@163.com, Phone and Fax: +86 02883037315*

## Experimental section

### Fabrication of the MgAl-LDH/Sep membranes

Firstly, The Sep and MgAl-LDH were dissolved in ultrapure water to make a 0.6g/L and 0.5g/L suspension, respectively. Secondly, under continuous stirring, the mixtures were dispersed uniformly by sonicating for 30 min. Finally, the samples were filtered onto the surface of substrate in vacuum one by one to form the hierarchical structure membrane.

Table S1 Compositions of the membranes prepared in this report

| Sample / Composition (mg) | LDH-M | Sep-M | M1 | M2 | M3 | M4 |
|---|---|---|---|---|---|---|
| LDH | 6 | 0 | 6 | 6 | 6 | 6 |
| Sep | 0 | 6 | 6 | 12 | 18 | 24 |
| $Al^{3+}$ | 750 | 750 | 750 | 750 | 750 | 750 |

## Oil–water emulsion separation and cycling test

The separation process was carried out on a vacuum apparatus. The O/W emulsion were poured onto the membranes and separated under vacuum filtrating. The cycling test corresponded to the anti-fouling ability of the membrane. For each separation cycle, 20 mL of an O/W emulsion was permeated through the membrane under a pressure of 0.1 MPa. The filtrate water was collected and the separation efficiency for each emulsion was calculated by TOC.

## Dye removal experiments

In this process, CR and MB as ionic dye were applied severally. Dye removal tests were performed through a filtration system with an effective membrane area of 12.566 $cm^2$. In detail, 20 mL of synthetic dye solution with diverse concentration was filtered, and then flux and rejection were determined using the equations of (1) and (2). Spectrophotometric measurement was used to obtain the dye concentration at 496 nm and 664 nm (UV–Vis spectrophotometer (UV-762 (Shanghai precision scientific instrument co.))). In addition, to exclude the randomness of the experiment and study the flux stability and pollution resistant capacity of all membranes, each experiment was conducted three times at 1.0 MPa. Furthermore, two dye solutions including 100, 200, 300 mg/mL CR and 10, 20, 30 mg/mL MB were tested on the membrane as anionic and cationic dyes, respectively.

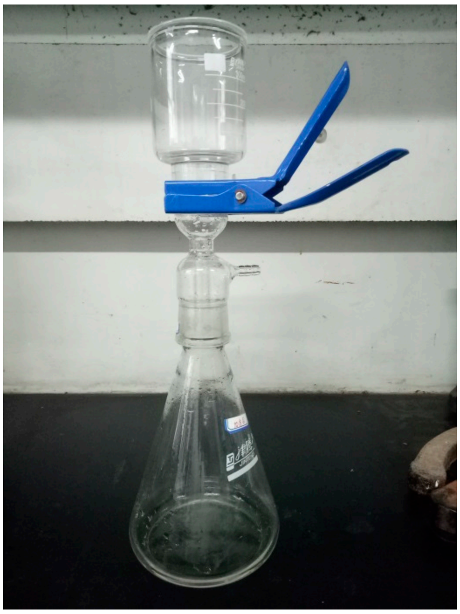

Figure S1. Filtering apparatus

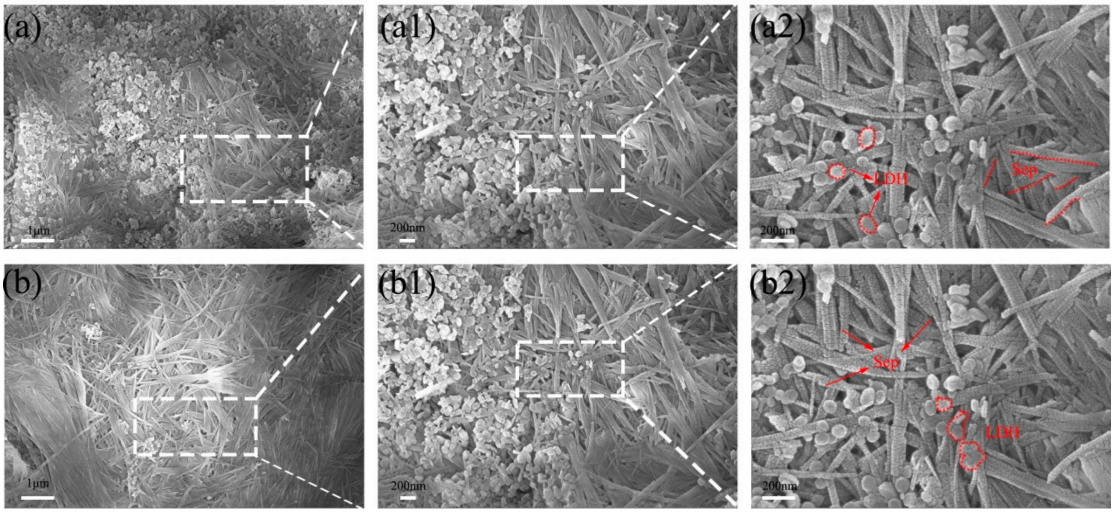


Figure S2. SEM image for M3 (a)-(a2) and M4 (b)-(b2) of membrane

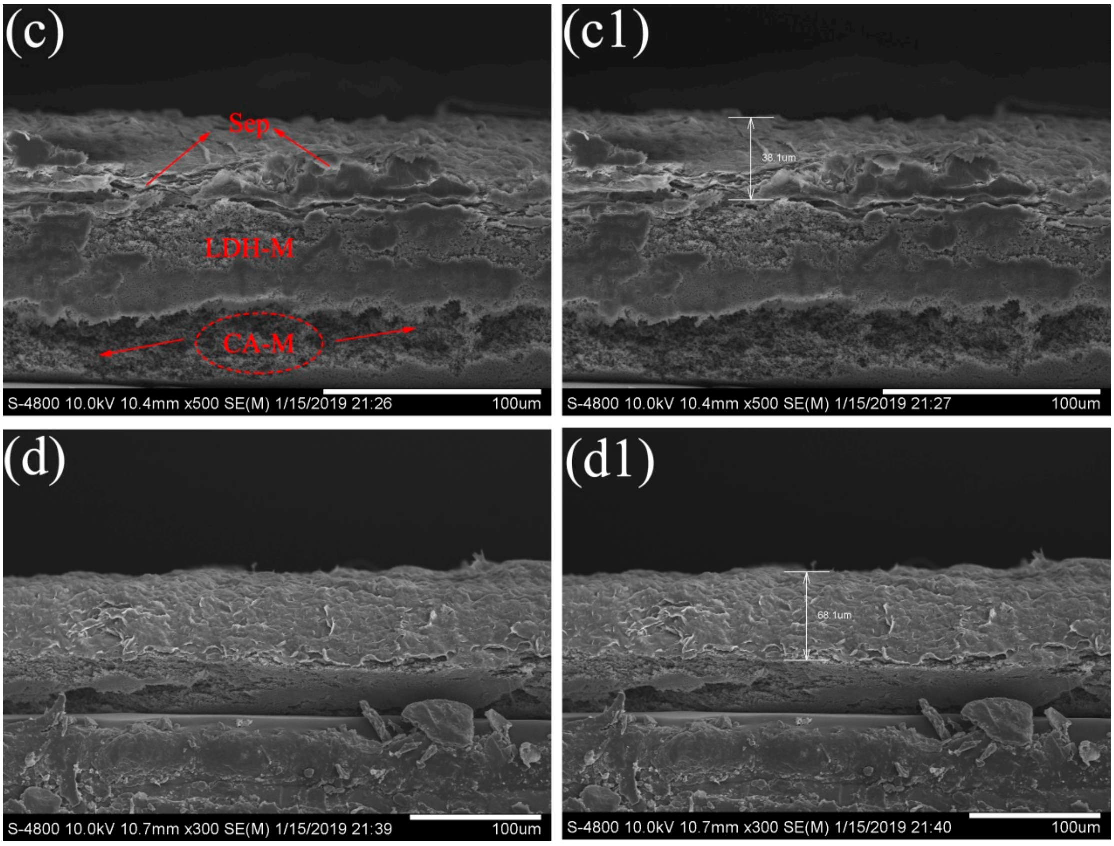


Figure S3. SEM image for M3 (c), (c1) and M4 (d), (d1) of membrane cross-section

Table S2 Surface roughness data

| Filter papers | Roughness | | Standard deviation(nm) |
|---|---|---|---|
| | $R_a$(nm) | $R_q$(nm) | |
| LDH-M | 72.1 | 106 | 27.6 |
| M2 | 166 | 217 | 47.2 |

Table S3 Pore size data

| Name | LDH-M | M2 |
|---|---|---|
| Measured bubble point pressure (bar) | 1.1026 | 3.8881 |
| Measured bubble point flow (L/min) | 0.0066 | 0.0041 |
| Minimum pore size pressure (bar) | 3.0256 | 15.135 |
| Average pore size pressure (bar) | 2.155 | 12.7582 |
| Gas permeability ($m^3/(m^2.pa.s)$) | 3.94E-02 | 7.09E-03 |
| Gas flux ($m^3/(m^2.h)$) ($\Delta P$=0.1000bar) | 1.45E+02 | 1.20E+01 |
| Measured bubble point pore size (μm) | 0.4367 | 0.1239 |
| Optimal pore size (μm) | 0.2278 | 0.0386 |
| Minimum pore size (μm) | 0.1592 | 0.0318 |
| Average pore size (μm) | 0.2235 | 0.0377 |

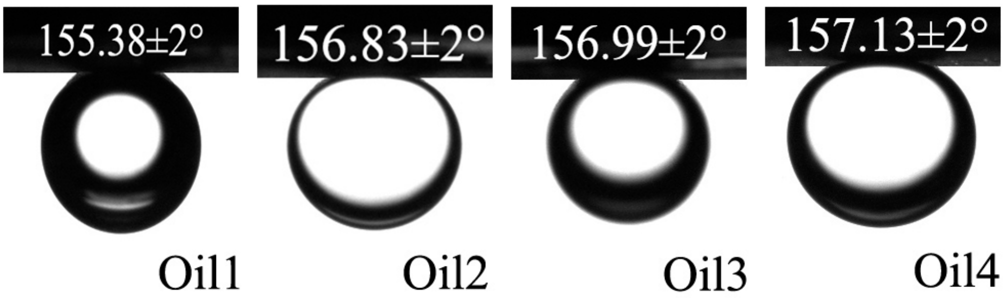


Figure S4. Underwater oil contact angle

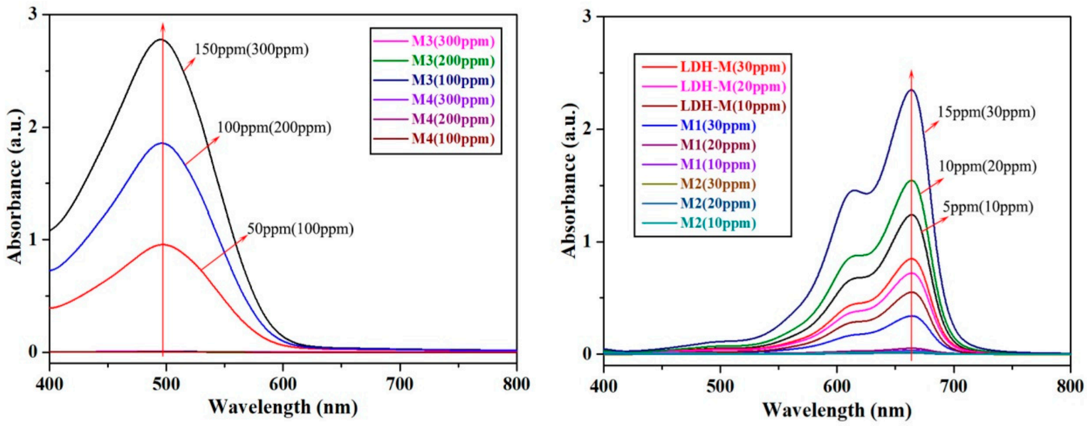


Figure S5. UV–visible absorption spectra

Table S4. The rejection of as-prepared membrane (M2) for different type of organic dye.

| Dye name | Molecular formula | Relative molecular mass (g/mol) | Volume (ml) | Concentration (ppm) | Rejection |
|---|---|---|---|---|---|
| Methylene Blue (MB) | 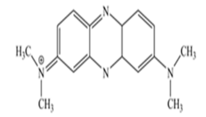 | 374 | 20 | 30 | 99.79% |
| Congo Red (CR) | 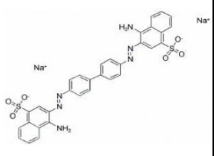 | 696.68 | 20 | 300 | 99.89% |
| Methyl Orange (MO) | 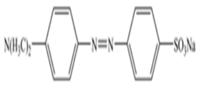 | 327 | 20 | 30 | 99.82% |
| Acid Red 87 (AR) | 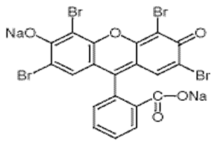 | 691.86 | 20 | 300 | 99.91% |

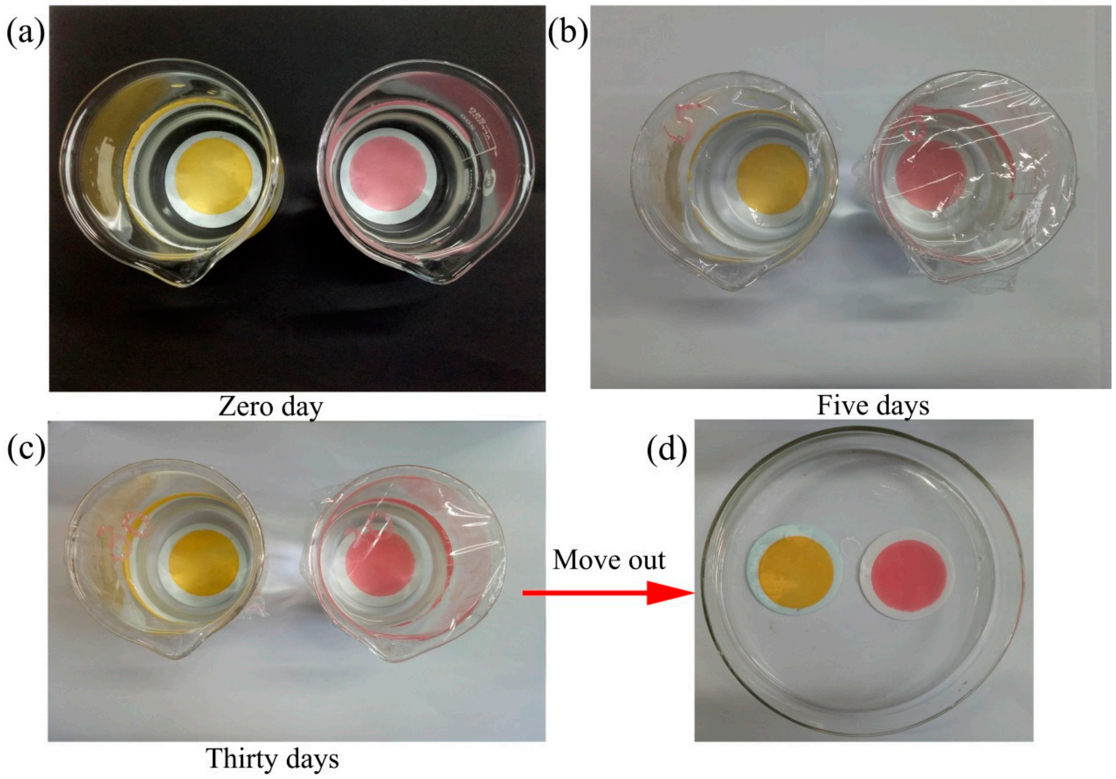


Figure S6.The photographs of the membrane in the water for 30 days (Since the color of the MgAl-LDH/Sep membranes is light yellow and difficult to observe, the membrane is dyed with a small amount of dye for easy observation.)
